# Incomplete compensation without further adaptation: A large-sample acoustic analysis of bite block speech in masking noise

**DOI:** 10.1121/10.0044347

**Published:** 2026-07-21

**Authors:** Timothy K. Murphy, Hung-Shao Cheng, Caroline A. Niziolek, Benjamin Parrell

**Affiliations:** 1 Department of Otolaryngology-Head and Neck Surgery, University of Wisconsin–Madison, 600 Highland Ave, Madison, Wisconsin 53792, USA; 2 Waisman Center, University of Wisconsin–Madison, 1500 Highland Ave, Madison, Wisconsin 53705, USA; 3 Department of Communication Sciences and Disorders, University of Wisconsin–Madison, 1975 Willow Dr, Madison, Wisconsin 53706, USA

## Abstract

The speech system maintains intelligibility even when articulatory movements are restricted. Researchers often study this compensatory mechanism using a bite block to constrain jaw movement. While early reports indicated that speakers fully negate bite block effects on vowel acoustics, more recent reports found only partial compensation, suggesting the potential for continual adjustments (adaptation). We investigated a large sample (*n* = 55) of speakers who performed a bite block task while their auditory feedback was masked. We found incomplete immediate compensation and no adjustments over time, suggesting that adaptation for bite block effects on vowel acoustics requires auditory feedback.

## 1. Introduction

When articulatory movements are constrained, the speech system flexibly adjusts to maintain intended productions. For example, when the position of the jaw is fixed with a bite block, speakers are still able to produce intelligible speech and relatively accurate vowel acoustics through compensatory tongue movements. However, studies report conflicting evidence in regards to the specificity and extent of the adjustments talkers employ when the jaw is fixed by a bite block. Compounding this issue is a consistent reliance on small sample sizes (typically fewer than 10 talkers), which raises the question of whether these studies are sufficiently powered to detect such adjustments.

Early studies largely suggested that talkers immediately and completely compensate for bite blocks, producing vowels with formant frequencies that do not differ from those of unconstrained productions from the very first repetition.^1–3^ This led to the development of a dynamic model of compensation wherein inherently self-equilibrating tongue-jaw synergies automatically adjust to achieve target configurations regardless of disruptions, allowing speakers to rely on feedforward control rather than needing to derive independent articulator-specific compensatory movements through sensory feedback.^4,5^ Kelso and Tuller^6^ provided support for this model by demonstrating complete, immediate compensation to a bite block despite the elimination of proprioceptive feedback (temporomandibular joint anesthetization), tactile feedback (topical oral anesthetic), and auditory feedback (masking noise). However, this study included only a total of five participants. Subsequent studies have reported that immediate compensation is, instead, incomplete, with initial vowel and consonant productions made just after the insertion of a bite block differing significantly from unconstrained productions.^7–10^ Of note is a study by McFarland and Baum,^9^ which included a somewhat larger sample of 15 talkers. In addition to finding significant differences in formants between baseline and initial bite block productions, they showed that speakers' vowel formants shifted toward baseline values after 15 minutes of speaking. These and similar findings were taken as evidence that auditory and somatosensory feedback can drive adaptation to compensate for the articulatory disturbance of the bite block.^7–9^ More recently, Zandipour *et al.*^10^ demonstrated, in a single-participant study, that when auditory feedback is masked, initial deviations in formant production after insertion of a bite block still moved toward baseline values over time, suggesting that somatosensory feedback alone is sufficient to guide adaptation. However, the generalizability of these findings beyond this single talker is difficult to assess. This lack of consensus regarding actual talker behavior during bite block speech stems largely from a methodological limitation: although numerous studies have used bite block data to argue for specific speech control mechanisms, the field has suffered from a historical reliance on very small sample sizes. To rectify this, the current study leverages a sample of 55 talkers to assess changes in vowel production caused by a bite block in the absence of auditory feedback, including both immediate compensation and further adaptation. First, we examine whether immediate compensation for a bite block in the presence of auditory masking noise is complete or incomplete by testing whether the acoustics of initial vowel productions after bite block insertion differ significantly from baseline, unconstrained productions. Second, we investigate whether speakers adapt their vowel productions toward unconstrained formant values over repeated productions by testing whether productions made after 15 repetitions with a bite block are closer to baseline productions than productions made just after bite block insertion. Finally, we perform two exploratory analyses to determine other effects of the bite block on speech motor control for vowels, examining changes in vowel duration and formant variability.

## 2. Methods

### 2.1 Participants

Sixty-two paricipants (48F/14M) between 18 and 78 years old (M = 39.3, SD = 19.3) completed the bite block task. Participants self-identified as native speakers of English with no reported history of neurological, speech, or hearing impairments. Hearing eligibility was confirmed via Hughson-Westlake pure-tone audiometry. Inclusion required thresholds ≤25 decibels hearing level (dB HL, 250–4000 Hz) for ages 18–55, and ≤40 dB HL (250–2000 Hz) with ≤55 dB HL at 4000 Hz for ages ≥56. Informed written consent was obtained before participation and participants received either monetary compensation or course credit. Experimental procedures were approved by the Institutional Review Board at the University of Wisconsin–Madison (IRB 2017-1128-CR008).

We excluded a total of seven participants from data analysis: five produced incorrect words (i.e., said a word other than the prompt) on trials that were critical for hypothesis testing, one received bite blocks of incorrect sizes across the bite block conditions, and one had persistent breathy phonation, which prevented reliable identification of vowel boundaries and formant tracking. Our final sample thus consisted of productions from 55 participants (M = 37.2, SD = 18.8; 43F/12M).

### 2.2 Procedure

On each trial, a word was displayed orthographically on the computer screen for 1.7 s, with a randomly jittered interstimulus interval ranging from 0.75 to 1.5 s. Participants were instructed to read the word aloud when it appeared on the screen. Participants' speech was recorded with a tabletop Sennheiser MKE 600 microphone (Wedemark, Germany), digitized with a sampling rate of 16 kHz. On all trials, participants heard loud, speech-shaped masking noise (∼80 dB SPL) via Beyerdynamic DT 770 Pro headphones (Heilbronn, Germany) which prevented them from using auditory feedback to guide their speech production. The noise was generated by Audapter,^11^ and the amplitude of the noise was scaled in real-time by the short-term amplitude envelope of the participant's speech such that noise was only played while participants vocalized. This minimized, as much as possible, the potential effects of speaking in noise on vowel formant production.^12–14^

The experiment consisted of three phases. All participants first completed a single 32-trial baseline phase where they read aloud the four target words (*heed*, *who'd*, *had*, *hod*; respectively containing the vowels /i/, /u/, /æ/, and /ɑ/) eight times each with no bite block (i.e., with unconstrained jaw movements). Words in the baseline phase were randomly ordered within sets of four, with each word occurring once per group. Subsequently, participants moved on to two bite block phases consisting of 32 trials each. In one phase, they produced speech with the small bite block, whereas in the other, they spoke with the large bite block. The presentation order of these two bite block phases was counterbalanced across participants. In the small bite block phase, a horizontally placed tongue depressor stabilized the jaw at a relatively closed position (2-mm aperture), decreasing jaw displacement for low vowels (/æ/ and /ɑ/) that are typically produced with an open jaw configuration.^15^ Target words in this phase were *had* and *hod* (randomized order). In the large bite block phase, the tongue depressor was positioned vertically to create a 17.5-mm jaw aperture, increasing jaw displacement for high vowels (/i/ and /u/) that are typically produced with a closed jaw configuration. Target words in this phase were *heed* and *who'd* (randomized order). In both bite block phases, researchers confirmed that the block did not obstruct tongue movement. However, the length of the tongue depressor did cause some interference with lip movements in the large bite block phase, which may have affected lip rounding during /u/ production in *who'd*.

### 2.3 Acoustic analyses

Vowel formant frequencies were estimated in Praat^16^ using linear predictive coding. The linear predictive coding order and pre-emphasis settings were manually adjusted for each participant and vowel using wave_viewer,^15^ a matlab GUI interface for formant tracking using Praat. Vowel onsets and offsets within each trial were automatically set by the Montreal Forced Aligner;^17^ vowel boundaries were visually verified to ensure that onsets aligned with the beginning of clearly defined formant tracks for the first (F1) and second (F2) formants and that offsets corresponded to the point where the formant tracks ended and amplitude decreased. For each trial, a single F1 and F2 measure was calculated based on the average formant values measured from the middle 50% of the vowel. All frequency measurements were converted to mels to better approximate human auditory perception given the nonlinear frequency resolution of the auditory system.

These raw formant measures (in mels) were subsequently transformed to better capture changes in vowel production in the first two vowel formants (F1/F2) as well as a more holistic measure reflecting distance in the 2D Euclidean space defined by these two formants. To capture the magnitude and direction of acoustic change from baseline vowel productions along each formant dimension, formant frequencies (F1/F2) measured from productions made on each trial were normalized for each participant and vowel by subtracting the corresponding median formant frequencies during the baseline phase. Additionally, to capture the total magnitude of acoustic change across both formant dimensions simultaneously, the Euclidean distance (in mels) between the F1/F2 values for each vowel production and the median F1/F2 values from corresponding vowel productions in the baseline phase were measured. Note that this distance metric results in positive values for all trials, including those in the baseline phase. All statistical analyses and results are reported using these normalized values (F1, F2, Euclidean distance).

### 2.4 Statistical analyses

To test whether participants' immediate compensation for a bite block was complete or incomplete, the direction and magnitude of immediate compensation along individual formant frequencies were assessed by comparing normalized F1 and F2 values from the initial bite block trial against zero using two-tailed one-sample *t* tests. A similar test was conducted for the more holistic measure of Euclidean distance to the median F1/F2 values in the baseline phase, here using one-tailed paired *t* tests comparing this distance on the first trial of the bite block phase to the median distance of the corresponding trials in the baseline phase. All three tests were conducted separately for each vowel (3 measures × 4 vowels = 12 tests total), with Holm-Bonferroni correction applied to control the family-wise error rate.

To test whether adaptation to the bite block occurs over repeated productions, we examined whether formant frequencies measured from vowel productions in the bite block phases would move toward the median formant frequencies in the baseline phase over the course of the bite block condition. To do this, we measured how far the initial and final bite block productions deviated from baseline vowel productions by calculating the absolute difference between each vowel production at the initial and final timepoints and the median baseline values for F1, F2, and F1–F2 Euclidean distance. This approach specifically captured the magnitude of deviation from baseline vowel productions at each timepoint, with reduced values indicating adjustments toward baseline. Statistical analyses for F1 and F2 used a repeated-measures ANOVA (rmANOVA) that included within-subjects factors of formant (F1, F2), vowel (/æ/, /ɑ/, /i/, /u/), and timepoint (initial and final trials of the bite block phase), with all two-way and three-way interactions. Significant vowel × timepoint interactions were analyzed with follow-up, formant-specific ANOVAs (one per formant). Separately, we tested for changes in Euclidean distance with a rmANOVA that included within-subjects factors of vowel and timepoint, as well as their interaction. For both models, the degrees of freedom and p values were Greenhouse–Geisser corrected where the assumption of sphericity was violated and, where appropriate, *post hoc* pairwise comparisons with Tukey's Honestly Significant Difference correction were used to identify which (if any) vowels shifted toward baseline.

We also conducted exploratory analyses of the effects of the bite block on vowel production, examining vowel duration and trial-to-trial formant variability. Vowel duration was calculated as the median duration of each vowel (in milliseconds) within each phase and analyzed using an rmANOVA with factors of vowel (/æ/, /ɑ/, /i/, /u/) and phase (baseline, bite block), including their interaction. Production variability (in mels) was quantified as the standard deviations of F1 and F2 (separately) across repetitions of each vowel within each phase. Prior to this, F1 and F2 measured within each phase were detrended in matlab to remove gradual drifts over the phase, isolating short-term fluctuations from any possible overall change, such as movement towards the baseline values in the bite block phase (for analyses on non-detrended data, see the supplementary material). Variability was analyzed using a rmANOVA with factors of formant, vowel, and condition, including all interactions; significant three-way interactions were followed up with separate ANOVAs for each formant and Tukey-corrected pairwise comparisons as appropriate.

## 3. Results

**Fig. 1.**
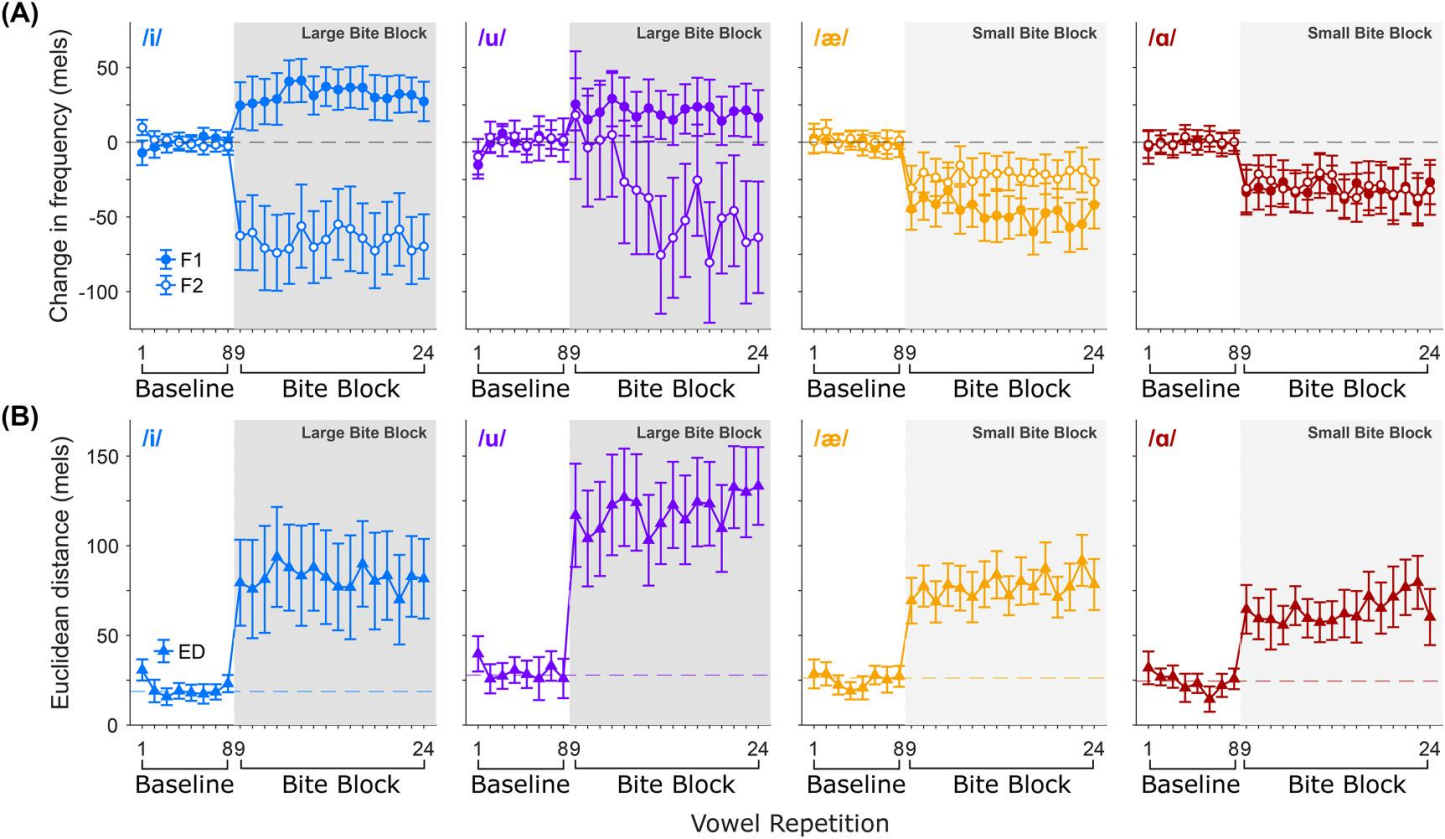
Change in vowel formants as a function of vowel repetition. (A) Change in F1 (solid circles) and F2 (open circles) relative to median formants of corresponding vowels in the baseline phase. (B) Euclidean distance in F1/F2 formant space relative to the median F1/F2 values in the baseline phase. Horizontal dashed line indicates average Euclidean distance in the baseline phase. Error bars represent 95% confidence intervals.

To determine whether participants' immediate compensation for the bite block was complete, we compared formants from the first vowel produced after bite block insertion with median values from productions in the baseline phase (Fig. 1; Table 1). Across all vowels and formant distance metrics, all but one showed significant differences between the initial bite block production and median baseline formant frequencies. For F1, all vowels showed significant changes (/i/, /æ/, *p_adj_* < 0.001; /u/, *p_adj_* = 0.002; /ɑ/, *p_adj_* = 0.034) with small to medium effect sizes (Cohen's *d* = 0.328–0.624); F1 increased in high vowels (/i/, /u/) and decreased in low vowels (/æ/, /ɑ/), consistent with the expected effects of the bite block on jaw position. For F2, significant decreases in frequency were observed for /i/, /æ/, and /ɑ/ (all *p_adj_* < 0.001) with medium to large effect sizes (Cohen's *d* = 0.535–0.989); conversely, /u/ showed a numerical increase in F2, though this was not significant (*p_adj_* = 0.056), likely due to much higher interspeaker variability in this measure. In agreement with these formant-specific results, all vowels showed an increase in Euclidean distance to the median baseline production between the baseline trials and the initial trial of the bite block phase (all corrected *p_adj_* < 0.001), with large effect sizes (Cohen's *d* = 0.892–1.037).

**Table 1.** Change in initial bite block production by vowel.

Vowel	Measure	Mean difference (mels)	SE	*d*	*t*	*p_adj_*
**/i/**	F1	37.278	7.942	0.624	4.694	<0.001
	F2	–86.851	11.680	–0.989	–7.436	<0.001
	ED	87.077	12.085	0.958	7.206	<0.001
**/u/**	F1	32.738	8.914	0.488	3.672	0.002
	F2	42.670	21.811	0.260	1.956	0.056
	ED	111.890	14.343	1.037	7.801	<0.001
**/æ/**	F1	–32.622	7.089	–0.612	–4.602	<0.001
	F2	–35.520	7.726	–0.611	–4.598	<0.001
	ED	49.176	6.807	0.961	7.225	<0.001
**/ɑ/**	F1	–18.869	7.646	–0.328	–2.468	0.034
	F2	–32.754	8.129	–0.536	–4.029	<0.001
	ED	47.538	7.081	0.893	6.713	<0.001

Having established that vowel production changes immediately after the introduction of a bite block, we then tested whether participants adapted to correct for these changes on the basis of somatosensory feedback alone (as auditory feedback was blocked by masking noise). To do so, we examined whether formant frequencies measured from vowel productions move toward the formant frequencies in the baseline phase over the course of the bite block phase. We found no evidence for such adaptation, with no change between the first and final productions of the bite block phase in F1, F2, or Euclidean distance (see the supplementary material). For formant-specific analyses, the main effect of formant (F(1, 54) = 35.379, p < 0.001, $\eta_{p}^{2}$ = 0.396), the main effect of vowel (F(1.306, 70.517) = 13.696, p < 0.001, $\eta_{p}^{2}$ = 0.202), and the two-way interaction between vowel and formant (F(1.306, 70.517) = 24.853, p < 0.001, $\eta_{p}^{2}$ = 0.315) were significant, suggesting that the change in vowel formants in the bite block phase (relative to the baseline phase) was both vowel- and formant-specific. Critically, however, neither the main effect of timepoint (F(1, 54) = 0.043, p = 0.836, $\eta_{p}^{2}$ =0.001) nor the three-way interaction among timepoint, vowel, and formant were significant (F(1.306, 70.517) = 0.649, p = 0.540, $\eta_{p}^{2}$ = 0.012), indicating that vowel formants were unchanged from the initial to the final trials of the bite block phase. Results from the analysis of Euclidean distance were similar: while there was a significant main effect of vowel (F(1.802, 97.300) = 15.997, p < 0.001, $\eta_{p}^{2}$ = 0.229), there were no significant effects of timepoint (F(1, 54) = 0.104, p = 0.748, $\eta_{p}^{2}$ = 0.002) or the two-way interaction between timepoint and vowel (F(1.802, 97.300) = 0.420, p = 0.689, $\eta_{p}^{2}$ = 0.008). To provide positive support for the lack of changeover the course of the bite block phase, we conducted follow-up Two One-Sided Tests^18^ (for methods and detailed results, see the supplementary material). This analysis showed that initial and final productions in the bite block phase were statistically equivalent across all measures for most vowels (*p_FDR_* < 0.05). The sole exception was /æ/, for which both the F1 and Euclidean distance were above the equivalence boundary (further from baseline) at the end of the bite block phase.

**Fig. 2.**
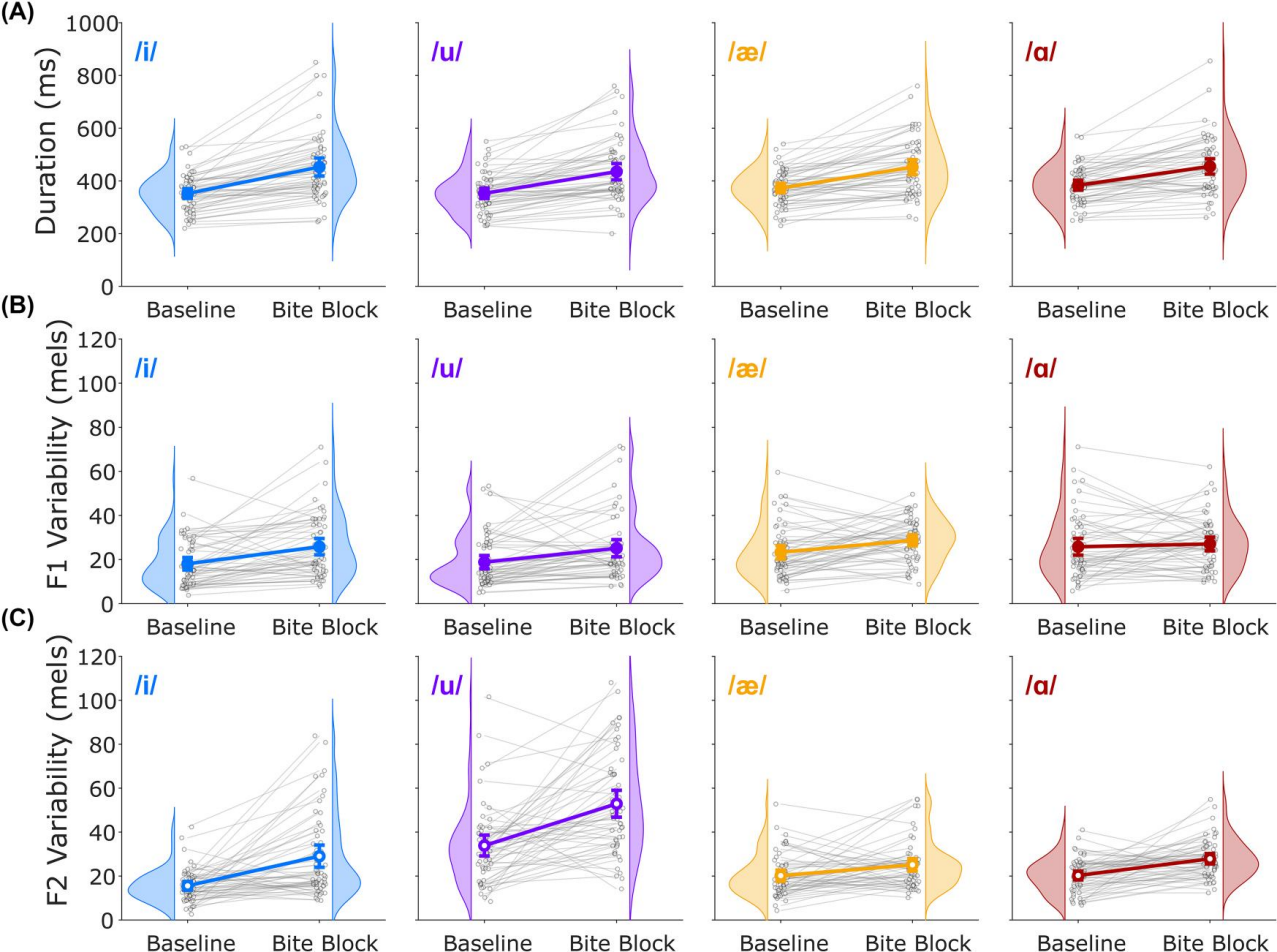
Effects of bite block on formant variability and vowel duration. Average vowel duration (A), standard deviation of F1 (B), and F2 (C) within phases. Gray markers represent individual participants; colored markers represent group means and 95% confidence intervals.

In addition to our primary analyses, we conducted two exploratory analyses examining the effects of bite blocks on vowel duration [Fig. 2(A)] and formant variability [Figs. 2(B) and 2(C)]. Overall, vowel duration increased in the bite block phase compared to the baseline phase (449 ± 2.1 ms vs 365 ± 1.3 ms; main effect of phase, F(1, 54) = 92.692, p < 0.001, $\eta_{p}^{2}$ = 0.632). Although individual vowels varied significantly in duration (F(1.153, 62.238) = 6.909, p = 0.002, $\eta_{p}^{2}$ = 0.113), duration increased in the bite block phase to a similar degree for all vowels (phase × vowel interaction, F(1.153, 62.238) = 2.869, p = 0.076, $\eta_{p}^{2}$ = 0.050; for details, see the supplementary material). For formant variability, vowel productions were generally more variable in the bite block phase than in the baseline phase for both F1 (5.2 mels increase) and for F2 (11.25 mels increase; main effect of phase, F(1,54) = 91.771, p < 0.001, $\eta_{p}^{2}$ = 0.630). As our omnibus test additionally showed significant effects of vowel formant as well as interactions between formant and the other factors (see the supplementary material), separate, planned, *post hoc* ANOVAs were conducted for F1 and F2 (see the supplementary material). These *post hoc* tests showed that the variability increase from the baseline to the bite block phase was significant for both F1 (F(1, 54) = 28.294, p < 0.001, $\eta_{p}^{2}$ = 0.344) and for F2 (F(1, 54) = 82.162, p < 0.001, $\eta_{p}^{2}$ = 0.603). This increase in variability was seen for all vowels, although there were vowel-specific differences in overall variability for both F1 (F(2.183, 117.864) = 5.565, p = 0.003, $\eta_{p}^{2}$ = 0.093) and F2 (F(1.806, 97.546) = 53.902, p < 0.001, $\eta_{p}^{2}$ = 0.500). For F2 only, the size of this increase varied between vowels (condition × vowel interaction, F2: (F(1.806, 97.546) = 6.629, p < 0.001, $\eta_{p}^{2}$ = 0.109), with high vowels showing larger increases in variability (/i/: 13.5 mels, /u/: 19.0 mels) than low vowels (/æ/: 4.9 mels, /ɑ/: 7.6 mels; for additional details, see the supplementary material).

## 4. Discussion

We leveraged a large sample of 55 talkers to assess the completeness of immediate compensation to a bite block in the absence of auditory feedback and to determine whether (in the case of incomplete compensation) adjustments continue over subsequent vowel repetitions. Two exploratory analyses tested whether the presence of the bite block affected other aspects of speech motor control, namely, vowel duration and formant variability.

Our first analysis provides strong evidence that immediate compensation is incomplete. Comparison of the initial bite block productions to median baseline formants revealed significant deviations across F1, F2, and Euclidean distances for all vowels, with the sole exception of a trend-level effect for /u/ (F2). Consistent (though vowel-specific) directional shifts in F1 and F2 were found across talkers, indicating systematic biasing of vowel productions by bite blocks that cannot be fully explained by individual variability in compensatory responses.

Demonstrating that compensation is systematically incomplete (in the absence of auditory feedback) allows us to reconcile conflicting evidence in the literature regarding the completeness of immediate compensation. Because the formant frequency changes caused by bite blocks are relatively subtle, studies with small sample sizes likely lack the statistical power to reliably detect them. For instance, a power analysis based on our median effect size (*d* = –0.536) indicates that a sample of *n* = 30 participants is necessary for a one-sample *t* test to detect a significant change in F1 or F2 with 0.80 power at the alpha = 0.05 level. This is much larger than the sample sizes in the literature, and twice the size of even the relatively larger sample (*n* = 15) utilized by McFarland and Baum,^9^ who failed to detect a significant effect on low vowel formant production with a small bite block similar in size to the one used in the present study.

Beyond statistical power, it is possible that variability in the specific dimensions of the bite blocks used across studies also may contribute to inconsistent findings in the literature. For instance, McFarland and Baum^9^ investigated high vowels using a “large” block (22.5 mm) considerably larger than ours (17.5 mm); this difference in magnitude may explain why they found a significant increase in F2 for /u/, whereas we observed only a trend-level effect, though in the same direction (however, we did replicate their finding a significant F2 decrease for the other high vowel /i/). Alternatively, this discrepancy may be due to the tongue depressor used as the bite block in our experiment, which partially obstructed the lips (particularly in the large bite block condition), potentially affecting lip rounding in /u/ productions. Nevertheless, the bite blocks used here were of relatively similar sizes to those in other studies which found complete immediate compensation either with normal feedback^1^ or under sensory deprivation,^6^ suggesting that the divergent results are more likely due to the small sample sizes in previous work.

Our findings challenge the view that tongue-jaw synergies alone are sufficient to fully preserve vowel acoustics.^6^ Consistent directional shifts in F1 and F2 frequencies across subjects suggest that bite blocks may biomechanically constrain the articulators such that anatomical adjustments alone cannot neutralize acoustic disruptions.^1^ Alternatively, the bite block may impose a novel articulatory constraint, placing the vocal tract in a configuration for which the speaker lacks the learned motor strategies necessary to preserve articulatory or acoustic goals. Given the relatively long latency of ∼100–150 ms for auditory-feedback based motor corrections,^19,20^ we believe that the incomplete compensation here measured at the vowel midpoint would also likely be observed without masking noise. However, it may be possible that the incomplete compensation we see here might be restricted to the case where auditory feedback is unavailable, and might be improved or even eliminated when auditory feedback is available. Even in this case, such a result would not contradict our current finding that articulatory synergies alone fail to preserve normal articulation in the presence of a bite block, as articulatory synergies are not thought to be critically dependent on the auditory system (e.g., Refs. 5 and 6). It would, however, rule out that the incomplete compensation we observe is due to biomechanical or motor constraints that prevent full compensation.

Our observation of incomplete compensation immediately following bite block insertion raised the possibility that speakers might improve through continual adjustments over time. Although the literature generally identifies auditory feedback as the primary driver of such adaptation,^7–9,15^ Zandipour *et al.*^10^ observed improved production in the absence of auditory feedback (though in a sample of only one speaker), which they argued suggested that the somatosensory feedback during ongoing speech production is sufficient for the motor system to refine its commands and, consequently, resolve the acoustic errors introduced by the bite block.

However, we found no evidence of continual adjustment when auditory feedback was masked in our large sample; final productions in the bite block phase were not significantly closer to baseline values than the initial productions. Thus, we conclude that somatosensory feedback alone appears insufficient to fully neutralize the acoustic perturbations caused by a bite block, at least in the short timescale examined here. Note that our null findings do not refute the idea that somatosensory feedback can be used to refine motor commands: speakers can compensate for jaw perturbations without auditory feedback,^21,22^ and some individuals seem to prioritize somatosensory over auditory cues when both are perturbed.^23^ However, they are consistent with a body of research suggesting that the continual maintenance of vowel acoustics relies primarily on auditory feedback. For instance, post-lingually deaf cochlear implant users exhibit reduced vowel and consonant contrasts when feedback is disrupted, but expand these contrasts upon its restoration.^15,24–26^ In the context of a bite block, auditory feedback can be used to adjust for the (initially uncompensated) effects on vowel acoustics,^9^ even in the complete absence of oral sensation.^27^

Our exploratory analyses demonstrate that the impact of bite block perturbations on vowel production extends beyond directional shifts in produced formant frequencies. First, we observed increases in vowel durations during bite block productions, in line with previous reports.^7,9,27,28^ These durational increases may reflect a mechanical limitation imposed by the bite block which necessitates an increase in the amount of time needed to move an articulator, such as the tongue.^28^ Alternatively (or additionally), increased vowel duration may reflect a compensatory strategy in which speakers slow down to ensure more accurate articulatory or acoustic accuracy,^7,29^ perhaps by relying more on sensory feedback to guide production.^30^ Second, we observed increases in F1 and F2 variability across all vowels. These results mirror findings from Lane *et al.*^15^ who found that the bite block increased vowel dispersion in F1/F2 space around vowel means while simultaneously reducing overall vowel space area. Increased vowel dispersion likely results from the bite block fixing the jaw, forcing the tongue to compensate with novel, unpracticed movements.

## 5. Conclusion

The current study provides strong evidence that adaptation to a bite block during vowel productions, in the absence of auditory feedback, is incomplete both immediately and over time. Our analysis of a large sample of talkers suggests that previous reports of complete immediate compensation in vowel production are likely due to low statistical power, though some specific differences in bite block size may explain some minor differences. Our results suggest that somatosensory feedback alone is not sufficient for driving adaptation to vowel formants perturbed by a bite block; while somatosensory feedback clearly allows participants to compensate to a large degree, but not completely, for this perturbation, auditory feedback appears necessary for speakers to further adapt their productions to achieve vowel formant values that are acoustically equivalent to their unperturbed productions.

## Supplementary Material

See supplementary material for tables containing statistical results for exploratory analyses.

## Data Availability

The data that support the findings of this study are openly available in the Open Science Framework at https://osf.io/kzhtm/.

## References

[1] C. A. Fowler and M. T. Turvey, “Immediate compensation in bite block speech,” Phonetica 37(5), 306–326 (1980). 10.1159/000260000 7280033

[2] T. Gay, B. Lindblom, and J. Lubker, “Production of bite block vowels: Acoustic equivalence by selective compensation,” J. Acoust. Soc. Am. 69(3), 802–810 (1981). 10.1121/1.385591 7240561

[3] B. Lindblom, J. Lubker, and T. Gay, “Formant frequencies of some fixed-mandible vowels and a model of speech motor programming by predictive simulation,” J Phon. 7(2), 147–161 (1979). 10.1016/S0095-4470(19)31046-0

[4] J. S. Kelso, B. Tuller, E. Vatikiotis-Bateson, and C. A. Fowler, “Functionally specific articulatory cooperation following jaw perturbations during speech: Evidence for coordinative structures,” J. Exp. Psychol. Hum. Percept. Perform. 10(6), 812–832 (1984). 10.1037/0096-1523.10.6.812 6239907

[5] E. Saltzman, “Task dynamic coordination of the speech articulators: A preliminary model,” Exp. Brain. Res. Ser. 15, 129–144 (1986). 10.1007/978-3-642-71476-4_10

[6] J. A. S. Kelso and B. Tuller, “Compensatory articulation under conditions of reduced afferent information: A dynamic formulation,” J. Speech Lang. Hear. Res. 26(2), 217–224 (1983). 10.1044/jshr.2602.217 6887808

[7] S. R. Baum, D. H. McFarland, and M. Diab, “Compensation to articulatory perturbation: Perceptual data,” J. Acoust. Soc. Am. 99(6), 3791–3794 (1996). 10.1121/1.414996 8655810

[8] J. E. Flege, S. G. Fletcher, and A. Homiedan, “Compensating for a bite block in /s/ and /t/ production: Palatographic, acoustic, and perceptual data,” J. Acoust. Soc. Am. 83(1), 212–228 (1988). 10.1121/1.396424 3343441

[9] D. H. McFarland and S. R. Baum, “Incomplete compensation to articulatory perturbation,” J. Acoust. Soc. Am. 97(3), 1865–1873 (1995). 10.1121/1.412060 7699168

[10] M. Zandipour, J. Perkell, F. Guenther, M. Tiede, K. Honda, and E. Murano, “Speaking with a bite block: Data and modeling,” in *Proceedings of the 7th International Seminar on Speech Production* (2006), pp. 361–368.

[11] S. Cai, S. S. Ghosh, F. H. Guenther, and J. S. Perkell, “Focal manipulations of formant trajectories reveal a role of auditory feedback in the online control of both within-syllable and between-syllable speech timing,” J. Neurosci. 31(45), 16483–16490 (2011). 10.1523/JNEUROSCI.3653-11.2011 22072698 PMC3268045

[12] E. Lombard, “Le signe de televation de la voix,” Annu. Mal. Oreille Larynx Nez Pharynx 27, 101–119 (1911).

[13] W. V. Summers, K. Johnson, D. B. Pisoni, and R. H. Bernacki, “An addendum to ‘Effects of noise on speech production: Acoustic and perceptual analyses’ [J. Acoust. Soc. Am. 86(4), 917–928 (1988)],” J. Acoust. Soc. Am. 86(5), 1717–1721 (1989). 10.1121/1.398602 2808921 PMC3521161

[14] W. V. Summers, D. B. Pisoni, R. H. Bernacki, R. I. Pedlow, and M. A. Stokes, “Effects of noise on speech production: Acoustic and perceptual analyses,” J. Acoust. Soc. Am. 84(3), 917–928 (1988). 10.1121/1.396660 3183209 PMC3507387

[15] H. Lane, M. Denny, F. H. Guenther, L. L. Matthies, L. Menard, J. S. Perkell, E. Stockmann, M. Tiede, J. Vick, and M. Zandipour, “Effects of bite blocks and hearing status on vowel production,” J. Acoust. Soc. Am. 118(3), 1636–1646 (2005). 10.1121/1.2001527 16240823

[16] P. Boersma and D. Weenik, “Praat: Doing phonetics by computer” [computer program], version 6.4.44, https://praat.org (Last viewed September 25, 2025).

[17] M. McAuliffe, M. Socolof, S. Mihuc, M. Wagner, and M. Sonderegger, “Montreal Forced Aligner: Trainable Text-Speech Alignment Using Kaldi,” in *Interspeech 2017*, pp. 498–502. https://www.isca-archive.org/interspeech_2017/mcauliffe17_interspeech.html (Last viewed March 20, 2026).

[18] D. J. Schuirmann, “A comparison of the two one-sided tests procedure and the power approach for assessing the equivalence of average bioavailability,” J. Pharmacokinet. Biopharm. 15(6), 657–680 (1987). 10.1007/BF01068419 3450848

[19] S. Cai, D. S. Beal, S. S. Ghosh, M. K. Tiede, F. H. Guenther, and J. S. Perkell, “Weak responses to auditory feedback perturbation during articulation in persons who stutter: Evidence for abnormal auditory-motor transformation,” PLoS One 7(7), e41830 (2012). 10.1371/journal.pone.0041830 22911857 PMC3402433

[20] B. Parrell, Z. Agnew, S. Nagarajan, J. Houde, and R. B. Ivry, “Impaired feedforward control and enhanced feedback control of speech in patients with cerebellar degeneration,” J. Neurosci. 37(38), 9249–9258 (2017). 10.1523/JNEUROSCI.3363-16.2017 28842410 PMC5607467

[21] S. M. Nasir and D. J. Ostry, “Somatosensory precision in speech production,” Curr. Biol. 16(19), 1918–1923 (2006). 10.1016/j.cub.2006.07.069 17027488

[22] S. Tremblay, D. M. Shiller, and D. J. Ostry, “Somatosensory basis of speech production,” Nature 423(6942), 866–869 (2003). 10.1038/nature01710 12815431

[23] D. R. Lametti, S. M. Nasir, and D. J. Ostry, “Sensory preference in speech production revealed by simultaneous alteration of auditory and somatosensory feedback,” J. Neurosci. 32(27), 9351–9358 (2012). 10.1523/JNEUROSCI.0404-12.2012 22764242 PMC3404292

[24] H. Lane, J. Wozniak, M. Matthies, M. Svirsky, and J. Perkell, “Phonemic resetting versus postural adjustments in the speech of cochlear implant users: An exploration of voice-onset time,” J. Acoust. Soc. Am. 98(6), 3096–3106 (1995). 10.1121/1.413798 8550935

[25] M. L. Matthies, M. Svirsky, J. Perkell, and H. Lane, “Acoustic and articulatory measures of sibilant production with and without auditory feedback from a cochlear implant,” J. Speech Lang. Hear. Res. 39(5), 936–946 (1996). 10.1044/jshr.3905.936 8898248

[26] M. A. Svirsky, H. Lane, J. S. Perkell, and J. Wozniak, “Effects of short-term auditory deprivation on speech production in adult cochlear implant users,” J. Acoust. Soc. Am. 92(3), 1284–1300 (1992). 10.1121/1.403923 1401516

[27] P. Hoole, “Bite block speech in the absence of oral sensibility,” in *Proceedings of the 11th International Congress of Phonetic Sciences* (1987), pp. 16–19.

[28] C. Dromey, M. Richins, and T. Low, “Kinematic and acoustic changes to vowels and diphthongs in bite block speech,” J. Speech Lang. Hear. Res. 64(6), 1794–1801 (2021). 10.1044/2021_JSLHR-20-00630 33979206

[29] S. J. Moon and B. Lindblom, “Interaction between duration, context, and speaking style in English stressed vowels,” J. Acoust. Soc. Am. 96(1), 40–55 (1994). 10.1121/1.410492

[30] S. G. Adams, G. Weismer, and R. D. Kent, “Speaking rate and speech movement velocity profiles,” J. Speech Lang. Hear. Res. 36(1), 41–54 (1993). 10.1044/jshr.3601.41 8450664

